# Barium concentration in cast roe deer antlers related to air pollution caused by burning of barium-enriched coals in southern Poland

**DOI:** 10.1007/s11356-016-6154-y

**Published:** 2016-01-28

**Authors:** M. Jabłońska, M. Kramarczyk, B. Smieja-Król, J. Janeczek

**Affiliations:** Faculty of Earth Sciences, University of Silesia, Będzińska 60, 41-200 Sosnowiec, Poland

**Keywords:** Cast antlers, Roe deer, Barium, Biomonitoring, Coal-burning

## Abstract

Concentrations of Ba, Zn, Pb, Fe, and Mn were determined by atomic absorption spectroscopy in freshly cast antlers from male roe deer of different ages (2 to 4 years old and older than 4 years) collected in Balin near Chrzanów and in the vicinity of Żywiec, S Poland. Barium content ranged from 124 to 196 ppm (mean 165 ppm) in the Balin 12 samples and from 207 to 351 ppm (mean 287 ppm) in 3 antlers from Żywiec. The concentration of Ba was comparable to that of Zn (134–275 ppm, mean 169 ppm). Elevated concentrations of Ba in antlers most probably originated from direct uptake of airborne barite nanocrystals through the respiratory system and/or by digestion of barite-rich dust particles deposited on plants. Burning of Ba-enriched coals is regarded as the principal source of Ba in the investigated areas inhabited by roe deer. Increased concentrations of Ba in antlers from the Żywiec area compared to Balin reflect particularly high air pollution caused by coal-burning mostly for domestic purposes combined with an unfavorable topography that impedes efficient air circulation.

## Introduction

Antlers are the only mammalian bony appendages capable of full regeneration and the fastest growing bones of vertebrates. Roe deer *(Capreolus capreolus)* initiate antler growth in autumn and antlers mature in early spring after ∼130 days, when the terminal mineralization of the antler bone stops. The skin (velvet) is shed and the so-called hard antlers are exposed. Antlers are retained on the buck’s head until autumn when they are cast followed by the immediate regeneration of a new set of antlers (Pielowski 1984; Kierdorf and Kierdorf 2005).

Antlers of various deer species are used as biomonitors for temporal and/or spatial variations in contaminant concentrations in the environment because during their growth they accumulate pollutants (Kierdorf and Kierdorf 2005). Strontium-90, a radionuclide with a half-life of ca. 29 years, was the first isotope studied in antler tissues to assess the environmental impact of radioactive fallout from atmospheric nuclear weapons tests (Hawthorn and Duckworth 1958). Fluoride and lead are the most commonly determined elements in antlers due to their preferred incorporation into the mineral phase of antler bone (e.g., Kardell and Källman 1986; Tataruch 1995; Kierdorf and Kierdorf 2002; 2003; 2005; Pokorny et al. 2004; Pokorny et al. 2009; Jelenko and Pokorny 2010; Sobota et al. 2011). Cd, Zn, Fe, Mn, Cu, Co, Cr, and Ni have also been determined in antlers (e.g., Sawicka-Kapusta 1978; Medvedev 1995; Purdey 2004; Landete-Castillejos et al. 2012). To our knowledge there are only two reports on Ba concentrations in antlers (Purdey 2004; Kierdorf et al. 2014).

The barium ion is toxic if bioavailable. For example, oral exposure to low-dose Ba can result in severe ototoxicity with degeneration of inner ears in mammals (Ohgami et al. 2012). Barium can potentially cause hypertension in humans (WHO World Health Organization 2004). Fortunately, Ba availability is largely limited in the environment due to the high stability of its main salt—barite (BaSO_4_; solubility product, K_sp_ of 1.08 × 10^−10^ at 25 °C). Still, under certain condition Ba can occur in soluble forms at concentrations much greater than expected from barite solubility (Pluta and Trembaczowski 2001; Smith et al. 2004; Smieja-Król and Bauerek 2015).

In the present study, the concentrations of Ba, Zn, Pb, Fe, and Mn were determined in roe deer antlers collected in two sites of southern Poland, located in the proximity of Upper Silesia – one of the European largest industrial and urban regions. Elevated concentrations of Zn, Pb, Cd, and Fe in antlers of roe deer were previously documented in Upper Silesian forests (Sawicka-Kapusta 1978). The purpose of this study was to determine whether the large-scale burning of Ba-enriched coals in Upper Silesia and surroundings is reflected in the Ba concentration in roe deer antlers.

## Materials and methods

Twelve freshly cast antlers from male roe deer of different ages (2 to 4 years old and older than 4 years) were collected 2–3 km west of a village (Balin) located between the cities of Jaworzno and Chrzanów, immediately to the east of Upper Silesia. Another three antlers from 2 to 4 year old male roe deer were collected in the forest of the Beskid Żywiecki mountains, 4–5 km south-east from the city of Żywiec. Most of the antlers were collected by local hunters near cultivated food plots in autumn 2009. Antlers overlooked during the autumn sampling campaign were found in spring 2010 after melting of snow cover. No animal was killed or harmed to collect antlers.

Atomic absorption spectroscopy (SOLAAR M6) was used for the determination of Ba, Fe, Mn, Pb, and Zn in antlers. Samples were homogenized in a mill, dried at 105 °C, then ashed at 550 °C for 24 h. The digestion was carried out in hot 5 % citric acid in platinum crucibles for 3–4 h. All metal concentrations were corrected for procedural blanks. The quality of the analytical data for Pb, Zn, and Fe was checked using the standard reference material (NIST 1486 bone meal). The results of metal analyses are presented on a dry weight basis. The limit of detection for Fe, Mn, Pb, and Zn in antlers was 2 ppm (mg/kg) and for Ba 20 ppm. Relationships between metals were examined using Spearman rank correlation coefficient at a confidence level *α* = 0.05.

## Results and discussion

### Metal concentrations in the antlers

The concentrations of Ba, Zn, Pb, Mn, and Fe in the analyzed antlers are given in Table 1. Barium content ranges from 124 to 196 ppm (mean value 165 ppm) in the Balin samples and from 207 to 351 ppm (mean value 287 ppm) in antlers from the vicinity of Żywiec (Tab. 1). The concentration of Ba is comparable to the concentration of Zn. The latter spans the range between 134 and 275 ppm, with a mean value of 169 ppm. Unlike Ba, there is no marked difference in Zn concentrations between samples from Balin and Żywiec. The concentration of Fe covers a wide range between 99 and 553 ppm. Lead concentration ranges from less than 2.0 ppm to 14.5 ppm. Manganese concentrations are scattered over a wide range (2.0–63 ppm), with an average value of 20.7 ppm. There is no obvious difference in the measured metals concentrations between deer of different age.

**Table 1:** Metal concentrations (ppm, dry weight basis) in roe deer antlers from Balin and the vicinity of Żywiec

Locality, deer age (years)	Ba	Zn	Pb	Fe	Mn
Balin, 2–4	144	166	<2.0	155	4.2
Balin, 2–4	178	175	<2.0	133	17.9
Balin, 2–4	196	275	4.2	183	43
Balin, 2–4	175	164	<2.0	171	42.2
Balin, 2–4	173	129	<2.0	106	5.8
Balin, >4	141	149	5.2	145	5.7
Balin, >4	167	170	<2.0	157	16.5
Balin, >4	124	162	<2.0	142	6.2
Balin, >4	150	154	<2.0	135	8.2
Balin, >4	166	154	<2.0	213	27.9
Balin, >4	182	187	12.3	553	17.5
Balin, >4	182	163	14.5	111	13.7
Żywiec, 2-4	207	202	6.5	425	63
Żywiec, 2-4	351	134	<2.0	99	37
Żywiec, 2-4	302	155	<2.0	97	2.0

Studies on the distribution of Ba in mammals, including humans, have shown that Ba accumulates preferably in bones over soft tissues. The typical Ba content in bones of terrestrial mammals is between 5–30 ppm, while concentrations in soft tissues range between 0.1 and 2.5 ppm (dry weight; Kabata-Pendias and Pendias 1999). The investigated antlers are on average (189 ppm) enriched in Ba over six times in respect to the highest reference concentration (30 ppm) in the mammalian bones provided by Kabata-Pendias and Pendias (1999). The higher content of Ba in antlers, relative to more slowly growing types of bone, can only be partially explained by the rapid incorporation of metals during antlers growth. The maximum Ba concentration of 351 ppm in antlers from the Żywiec area is markedly higher than the maximum value of 280 ppm obtained by Purdey (2004) in antlers of deer thriving in areas affected by chronic wasting decease. The maximum Ba concentration in antlers of North American deer living in areas not affected by chronic wasting decease was 74 ppm (Purdey 2004). High Ba concentrations (255–395 ppm, dry weight) were recorded in red deer antlers sampled in the 1980s and early 1990s in western Germany and the Netherlands (Kierdorf et al. 2014).

Concentrations of Pb (Table 1) are well within a range reported for antlers (4.8-28 ppm) by Sawicka-Kapusta (1978) and bone tissues (4–25 ppm; Kabata-Pendias and Pendias 1999), whereas the highest concentrations of Fe measured during this study (425 and 553 ppm) exceed Fe values typical for bones (5–300 ppm; Kabata-Pendias and Pendias 1999). Zinc preferentially concentrates in muscles (100–200 ppm) over bones (50–150 ppm) (Kabata-Pendias and Pendias 1999). Its average content in the antlers (169 ppm) is slightly above the bone range and similar to values (110–180 ppm) reported by Sawicka-Kapusta (1978) for antlers sampled in Upper Silesia. The Mn content in antlers ranges from 2 to 63 ppm and in six samples (Table 1) its content is 2 to 6 times higher than typically found in bones (0.2-10 ppm; Kabata-Pendias and Pendias 1999). The statistical analysis (Spearman rank correlation) revealed weak positive correlations only between Fe and Zn (*r*_s_ = 0.61; *p* < 0.05) and Fe and Mn (*r*_s_ = 0.53; *p* < 0.05), while concentrations of the other elements were uncorrelated (all *p* > 0.05).

### Sources of barium

The main source of barium in the antlers from Balin and Żywiec is most probably Upper Silesian coal-burning for both domestic and industrial uses (Różkowska and Ptak 1995; Wójcik and Smołka-Danielowska 2008; Wilczyński 2013). Processing of Zn-Pb ores and dust deposition from cement plants might be of some importance in Balin, although these activities have recently been significantly reduced.

Barium is a coalphile element with a coal affinity index (CAI = average element concentration in coal ash/Clarke value in sedimentary rocks) of 2.3 (Ketris and Yudovich 2009). Barium concentrations in Upper Silesian coals range from 0.2 to 4260 ppm with a mean value of 176 ppm (Różkowska and Ptak 1995), higher than the mean Ba concentration of 150 ppm in world coals (Ketris and Yudovich 2009). Accordingly, Ba content in the ash of Upper Silesian coals is almost twice as high (1852 ppm) as the world’s mean concentration of 980 ppm, resulting in a CAI of 4.5.

**Fig. 1:**
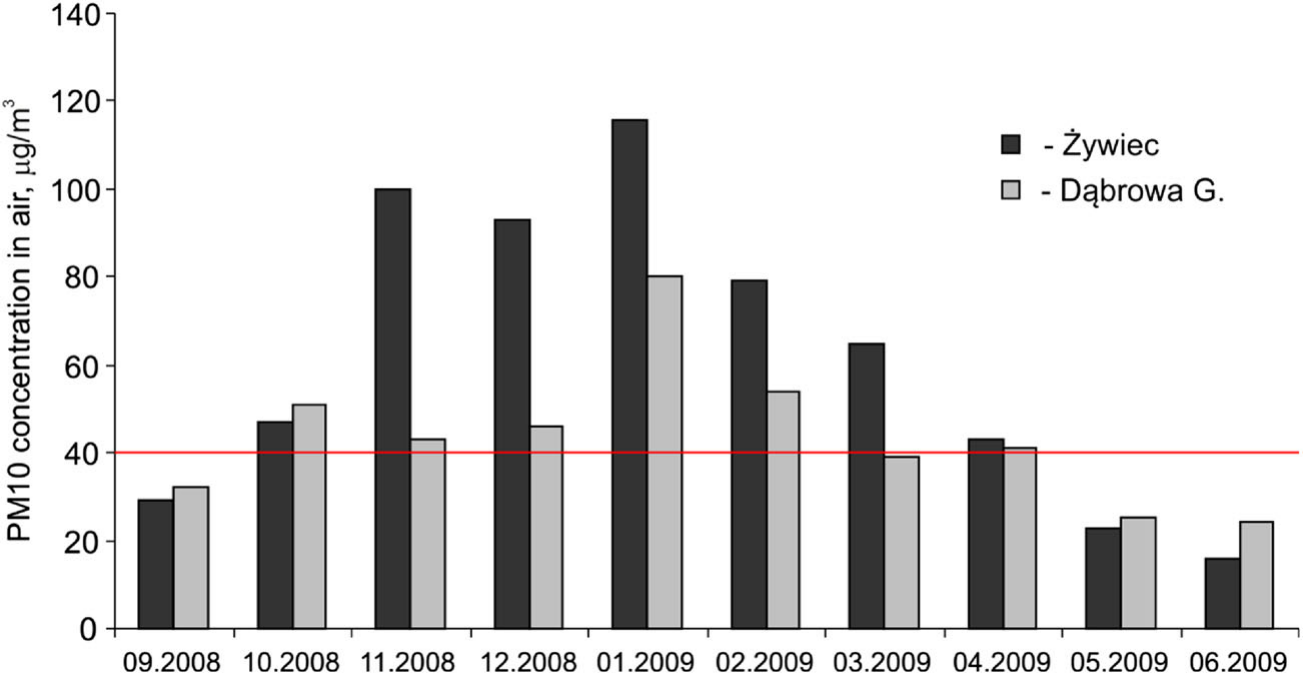
Concentration of airborne particulate matter with the aerodynamic diameter less than 10 μm (PM10) at monitoring stations located close to the sampling sites (http://monitoring.katowice.wios.gov.pl/)

In 2009, the dust emission from coal combustion in Poland amounted to 176,400 Mg. Sixty-six percent of that dust was emitted from domestic heating systems (Wilczyński 2013). As a result of such a high dust emission, the threshold value of 40 μg/m^3^ set by the EU for particulate matter (PM10) was significantly exceeded in the antlers’ collecting sites during the heating season (Fig. 1). Żywiec and its surroundings particularly suffered from the ineffective coal combustion in domestic heating systems while lacking large industrial emission sources. The problem of air pollution is worsened by the location of the city in a intermontane basin and the resulting impeded air circulation.

Barite nanocrystals (20 to 160 nm in diameter) are common and abundant in atmospheric dust in and around the region of Upper Silesia (Jablonska et al. 2001). Numerous of these nanocrystals occur within the respirable particles of hydrocarbons that can enter the mammalian respiratory system. In fact, barite particles, 0.42–0.60 μm in diameter, were observed in lungs of humans living in Upper Silesia (Jablonska 2013).

### Barium incorporation pathways

Two uptake routes must be considered to explain Ba accumulation in roe deer antlers, viz. direct uptake through the respiratory system and by digestion. Dermal absorption is not expected to play any significant role. The solubilization of inhaled barite has been demonstrated in numerous studies by clearance of barite from the respiratory system followed by skeletal accumulation and urinary excretion of Ba (e.g., Morrow et al. 1964; Cuddihy et al. 1974).

The exposure to barium through food ingestion can be of significance only if Ba-enriched dust particles are deposited on plant surfaces. Barite particles were observed on Scots pine (*Pinus sylvestris*) needles by scanning electron microscopy (Teper 2009) as result of wind re-deposition from Zn-Pb flotation tailing ponds at a site located 17 km NE from Balin.

Barium concentration in soils and its uptake by plants seem to be less important for the Ba accumulation in antlers. Barium concentrations in antlers from the Żywiec area are higher than in antlers from Balin (Table 1), despite the higher Ba content in soils near Balin. Barium concentration in topsoil (0–30 cm) in Balin ranges from 30 to 240 ppm (Pasieczna 2011); whereas Ba content in topsoil in the vicinity of Żywiec is generally less than 50 ppm (Lis and Pasieczna 1995). While plants are known to accumulate Ba in their green parts (Kabata-Pendias and Pendias 1999; Gałuszka et al. 2015), and food ingestion was identified as the primary exposure to Ba (ATSDR Agency for Toxic Substances and Disease Registry 2007), this intake route cannot explain the difference in Ba concentrations in antlers from the Balin and Żywiec areas.

## Conclusions

Elevated concentrations of Ba observed in antlers during this study most probably originated from direct uptake of airborne barite nanocrystals through the respiratory system and/or by digestion of barite-rich dust particles deposited on plants. Burning of Ba-enriched coals is the principal source of Ba in the investigated areas inhabited by roe deer. It is not known if elevated concentrations of Ba in antlers reflect adverse health effects caused by accumulation of this toxic element in deer as suggested by studies in North America. The mechanism of solubilization of apparently insoluble barite inhaled or ingested by mammals requires further study.
